# Knowledge about cataract and associated factors among adults in Gondar town, northwest Ethiopia

**DOI:** 10.1371/journal.pone.0215809

**Published:** 2019-04-23

**Authors:** Yezinsh Addis Alimaw, Mohammed Seid Hussen, Tsehay Kassa Tefera, Betelhem Temesgen Yibekal

**Affiliations:** Department of Optometry, School of Medicine, College of Medicine and Health Science, University of Gondar, Gondar Town, Ethiopia; Edith Wolfson Medical Center, ISRAEL

## Abstract

**Purpose:**

The aim of this study was to assess knowledge regarding cataract and associated factors among adults in Gondar town Northwest Ethiopia.

**Methods:**

A community-based cross-sectional study was conducted on 836 adults age ≥18years, using multi-stage systematic random sampling technique, in Gondar town Northwest Ethiopia from April 15-May 7, 2017. Data were collected using pre-tested structured questionnaires through face to face interview. The collected data was entered to Epi info version 7 and analyzed using SPSS version 20. Binary logistic regression was used to identify associated factors. Those variables with p-value <0.05 and confidence interval ≠ 1 in multivariable logistic regression were considered as statistically significant factors for knowledge regarding cataract.

**Result:**

Among 845 eligible adults, 98.9% (836) of them were fully participated. The median age of participants was 28 years with an interquartile range of 17 years. Of the total participants, 67.2% (562) of them had awareness about cataract [95% CI, 63.8–70.2]. Among 562 participants, 61.7% of them had good knowledge about cataract [95% CI, 57.5–66.00]. It was also found that higher level of education [AOR = 2.86, 95%CI: 1.37–5.96], higher family monthly income [AOR = 1.92, 95%CI: 1.03–3.57], having previous eye examination [AOR = 1.53, 95% CI: 1.02–2.31] and positive family history of cataract [AOR = 1.76, 95%CI: 1.03–3.01] were positively associated with good knowledge.

**Conclusion:**

Significant portion of the participants had good knowledge about cataract, which was positively associated with higher level of education, higher family monthly income, presence of previous eye examination and positive family history of cataract. However, significant knowledge gap regarding the risk factors and prevention strategies was recognized. Hence, it might be logical to pay special attention in prospering knowledge on how to prevent the occurrence of the disease.

## Introduction

Cataract is the opacity of the natural human lens, which may be resulted from congenital, developmental and acquired causes. Cataract is the leading causes of blindness worldwide, which accounts about more than half of 39 million blind people worldwide, and its blindness effect increases particularly in Sub Saharan Africa [1–4]. In Ethiopia 50% of blindness is caused by cataract [5]. It affects all age groups even though it is highly prevalent in people’s age greater 50 years [4, 6–8]. It is the most avoidable condition if timely intervention is instituted. Otherwise, it results in different catastrophic complications that end up with irreversible blindness [9]. As a result, its negative psycho-social economic impact is manifested at individual, family and community level [10, 11].

Several studies showed that the reasons for delaying from timely treatment are low economic status, lack of transportation, wrong perception, residual vision and poor knowledge (about the risk factors, nature of disease and treatment options) [4, 9, 12, 13]. Knowledge about cataract is the most vital aspect for delaying the occurrence of cataract, to initiate regular eye checkup, and to institute timely intervention. This, in turn, reduces the burden of the disease [12, 14, 15]. Furthermore, assessing knowledge regarding cataract is a precondition for designing health education and promotion programs [15–17]. In developing countries, it is conceived that health education and promotion plays a significant role in reducing burden of avoidable causes of blindness and visual impairment [15–19].

Several previous studies revealed that there was a gap in knowledge regarding cataract in developing and some developed countries. The studies also considered age, literacy, residency, marital status, previous exposure for eye care services, and other socio-economic variable as determinant for knowledge concerning to cataract [9, 12, 17–23]. Although there are different types of cataract which can affect all adult population, most of previous studies conducted on age-related cataract and consider subjects age greater than 40 years. In addition, majority of previous studies were institution based. Hence, the findings were less generalizable to all adult population. So, community-based study on knowledge about cataract was limited in Ethiopia. Therefore, this study aimed to assess the general knowledge of adult population regarding cataract and its associated factors, which will provide basic information for researchers, policymakers and resource allocators to plan health education and promotion programs to allow disease prevention and take early treatment options.

## Methods and materials

### Study design and period

A community -based cross-sectional study was conducted among adults in Gondar town from April 15 –May 7, 2017.

### Study setting

Gondar town is located 748 km from the capital city, Addis Ababa. There is one tertiary eye care center which provides different specialty eye care services and training of eye care professionals such as Optometrists, Ophthalmologists, and ophthalmic nurses.

### Sample size and sampling method

A total of 845 sample size was determined using single population proportion formula by assuming 95% confidence level, 5% margin of error, 50% proportion of good knowledge about cataract, design effect of 2 and 10% non-response rate. Since multi-stage systematic random sampling was used design effect was considered to minimize sampling error and assure representativeness. During sampling, First, 5 kebeles were selected randomly using a lottery method from the total of 21 kebeles of Gondar town. There were 18,542 households in the selected kebeles and the total adult populations in the selected households were approximately 37,084 adults [24]. Then the computed sample size was proportionally allocated for the selected kebeles and systematic random sampling was applied to select participant’s household with sampling fraction (K) of 21. Finally, one adult was selected from each household as a study participant. Lottery method was used if the number of adults were two or more per household. All adults aged ≥18 years had equally likely chance to participate in this survey. Nevertheless, those adults who had mental illness were excluded.

### Operational definitions

Participants’ **Knowledge about cataract** was assessed using 12 questions that consisted of six domains such as simple description, risk factors, symptoms, complications, treatment options and prevention strategies. Each item was equally weighted. Thus, each correct response had a score of 1 and each wrong response or do not know had a score of 0. Hence, the aggregate score for all knowledge questions would range from 0–12 points. Participants’ overall knowledge was categorized using the mean score, as **good** if the score was between 6–12 points and **poor** if the score was less than 6 points.

### Data collection tools and personnel

The data was collected using a pretested structured questionnaire. The tool consisted of socio-demographic and economic items, previous eye examination and knowledge about cataract. The questions concerning knowledge about cataract with respect to definition, risk factors, symptoms, complications, treatment option and prevention strategies were derived after literature review. First, the domain of the questionnaire was identified based on the objective of the study. Then the items were formatted in which some of them were open-ended and others were closed ended questions. Mover, the questions were arranged in logical sequence. Secondly, the original questionnaire was translated from English to Amharic version and then translated back to English by two independent local language translators to maintain its consistency and accuracy. Thirdly, before the actual data collection, pretest was performed on 5% of the sample to ensure the understandability of the items. Based on the pre-test, discussion was held with data collectors to compile the feedback and to modify as per necessary. Fourthly, the content of questionnaire with respect to clearness, easiness, included domains; reproducibility was validated by experts from Optometry department. Finally, the data was collected by trained 8 BSC optometrists through face to face questionnaire interview in the home to home visits at weekend.

### Data processing and analysis

After the collected data were checked for completeness and consistency, it entered into EPI INFO version 3.5.1 and exported to SPSS version 20 for analysis. The descriptive data was summarized by summary statistics such as mean, median, standard deviation and interquartile range. The model was checked using Hosmer- Lemeshow goodness of fit and Bivariable and multivariable binary logistic regression were used to identify associated factors for knowledge about cataract [25]. Those variables with a p-value of < 0.05 in multivariable binary logistic regression were considered as statistically significant. Finally, the analyzed result was presented using tables and charts.

### Ethical consideration

The study was conducted in accordance with the Declaration of Helsinki and approved by the University of Gondar Ethical Review Board. The purpose of the study was explained for each study subjects and verbal informed consent was obtained from each voluntary participant. As the study didn’t have an invasive procedure, the University of Gondar ethical review committee approved to use verbal informed consent to conduct this study. Health education about cataract was given after the interview for each study participant.

## Results

### Socio-demographic characteristics of the study participants

Among the total of 845 eligible adults, 836 of them completed the interview, which represents the response rate of 98.9% (836/845). Of the study participants, 64.8% (542/836) were females. The median age of study participants was 28 with an inter-quartile range of 17 years. The majority of respondents 74.5% (623/836) were young adults (18–29 years). Thirty-nine percent (326/836) of the study participants had the educational level of college/university. “Table 1”

**Table 1:** Socio-demographic characteristics of study participants at Gondar town, North West Ethiopia, 2017 (n = 836).

Variables	Frequency	Percent (%)
**Age(years)**		
**18–29**	623	74.50
**30–39**	82	9.80
**40–49**	51	6.10
**>50**	80	9.60
**Sex**		
**Female**	542	64.80
**Male**	294	35.20
**Religion**		
**Christian**	791	94.60
**Muslim**	45	5.40
**Marital status**		
**Married**	392	46.90
**Unmarried**	334	39.90
**Divorced**	44	5.30
**Widowed**	66	7.90
**Educational level**		
**Can’t read and write**	90	10.80
**Can read and write**	82	9.80
**Primary school**	113	13.50
**Secondary school**	225	26.90
**College/university**	326	39.00
**Occupation**		
**Government worker**	225	26.90
**Nongovernment worker**	192	23.00
**Student**	207	24.80
**Housewife**	201	24.00
**Retired**	11	1.30
**Family monthly Income (USD)**		
**<35**	250	29.90
**35–82**	199	23.80
**82–177**	256	30.60
**>177**	131	15.70

### Previous history of eye examination of the study participants

Among 836 respondents, 29.8% (249/836) of them underwent previous ocular examination. From those who had previous ocular examination, the majority of them 57.0% (142/249) underwent eye cheek up when they felt pain. Moreover, 69.1% (172/249) participants had last eye visit before 2 years. “Table 2”

**Table 2:** Previous history of eye examination of the study participants at Gondar town, North West Ethiopia, 2017.

Variables	Frequency	Percent (%)
**Previous eye examination(n = 836)**		
Yes	249	29.80
No	587	70.20
**Regularity of eye checkup (n = 249)**		
≤2 years	71	28.50
>2 years	36	14.50
When they feel pain	142	57.00
**Last eye visit(n = 249)**		
≤2 years	172	69.10
>2 years	77	30.90

### Participants’ knowledge regarding cataract

In this study, 67.2% (562/836) of the participants had heard of cataract [95% CI: 33.8% -70.2%]. The mean knowledge score point for those who had awareness was 6.02 ± 2.8 points. About 36.8% (207/562) of the participants reported that their main source of information was their family/friends followed by medical personnel 32% (180/562), television15% (85/562), radio13% (73/562) and magazine/books 3% (17/562).

Among 562 participants, 23.1% (130/562) of them gave a correct simple definition of cataract. Regarding risk factors of cataract, about 35% of the participants identified older age (201/562) and trauma as a risk factor (198/562). Forty-seven percent (262/562) of the participants recognized UV light as a risk factor for cataract. Eighty percent (451/562) of them conceived that cataract is a treatable condition and 67.8% (381/562) of the respondents cited surgery as the best treatment option. Forty percent 40.2% (226/562) participants stated that it is possible to prevent risk factors for cataract. However, only 18% (101/562) of participants mentioned some prevention mechanisms correctly “Table 3”. Overall among 562 participants, 61.7% of the participants had good knowledge about cataract [95% CI: 57.5%-66.0%].

**Table 3:** Participants’ knowledge about cataract in Gondar town North West Ethiopia, 2017(n = 562).

Questions	Responses		
	Correct n (%)	Incorrect n (%)	Don’t know n (%)
Definition of cataract	130(23.10)	290(51.60)	142(25.30)
Symptoms of cataract	366(65.12)	70(12.46)	126(22.42)
Age as a risk factor	201(35.76)	223(39.68)	138(24.56)
Age onset for ARC	333(59.25)	123(21.89)	106(18.86)
Trauma as a risk factor	198(35.23)	200(35.59)	164(29.18)
UV as a risk factor	262(46.60)	101(18.00)	199(35.00)
Worst effect of cataract	345(61.40)	141(25.10)	76(13.50)
Treatability of cataract	451(80.25)	25(4.45)	86(15.30)
Best treatment option	381(67.79)	126(22.42)	55(9.79)
Reversibility of vision after treatment	380(67.60)	44(7.80)	138(24.60)
Prevention of risk factors	226(40.20)	117(20.80)	219(39.00)
Mechanism of prevention	101(17.97)	125(22.24)	336(59.79)

### Associated factors of knowledge about cataract

The result of multivariable binary logistic regression analysis showed that educational level, family monthly income, previous history of eye examination and family history of cataract were significantly associated with knowledge about cataract. Hence, those participants with an educational level of primary school had 2.4 times more likely to have good knowledge than those who can’t read and write [95%CI: 1.09–5.37]. Similarly, those participants whose educational level of high school and University/college were 2.3 [95%CI: 1.10–4.70] and 2.9 [95%CI: 1.38–5.97] times more likely knowledgeable as compared to those who can’t read and write, respectively.

Regarding monthly income, Participants with family monthly income ranged from 35-82USD were 2.2 times more likely to have good knowledge than those earned <35USD [95%CI: 1.33–3.67]. Similarly, participants with family monthly income between 82-177USD and >177USD were 1.8 [95%CI: 1.14–3.10] and 1.9 [95%CI: 1.03–3.57] times more likely to have good knowledge than those earned <35USD, respectively.

Those study participants who underwent previous eye examination were 1.5 times more likely to have good knowledge as compared to those who didn’t have previous eye examination [95%CI:1.02–2.31]. And those participants who had a family history of cataract were 1.8 times more knowledgeable than those with no family history [95%CI: 1.03–3.01]. “Table 4”

**Table 4:** Factors associated with knowledge about cataract among adults in Gondar town, Northwest Ethiopia, 2017(n = 562).

Variable	Knowledge		COR (95%CI)	AOR (95%CI)
	Poor	Good		
**Age in year**				
18–29	136	258	1.00	1.00
30–39	26	35	0.71(0.41–1.23)	0.78(0.41–1.50)
40–49	21	23	0.58(0.31–1.08)	0.77(0.36–1.67)
>50	32	31	0.51 (0.30–0.87)	0.75(0.34–1.67)
**Sex**				
Female	150	197	1.00	1.00
Male	65	150	1.76(1.23–2.52)	1.40(0.95–2.07)
**Marital status**				
Married	113	168	1.00	1.00
Unmarried	67	134	1.35(0.92–1.96)	1.08(0.69–1.67)
Divorced	8	22	1.85(0.79–4.30)	3.09(1.24–7.71)
Widowed	27	23	0.57(0.31–1.05)	1.16(0.54–2.52)
**Religion**				
Christian	205	328	1.00	1.00
Muslim	10	19	1.19(0.60–1.16)	1.68(0.72–3.91)
**Educational status**				
Can’t read and write	36	23	1.00	1.00
Can read and write	31	24	1.21(0.57–2.56)	1.20(0.55–2.64)
Primary school	23	40	2.723(1.31–5.66)	2.42(1.09–5.37)*
Secondary school	54	89	2.58(1.38–4.81)	2.27(1.10–4.70)*
College/university	71	171	3.77(2.09–6.81)	2.87(1.38–5.97)*
**Occupation**				
Government worker	51	132	2.16(0.63–7.38)	1.03(0.24–4.46)
Private worker	42	92	1.83(0.53–6.32)	1.42(0.35–5.87)
Student	45	53	0.98(0.28–3.43)	0.71(0.16–3.13)
Housewife	72	64	0.74(0.22–2.54)	0.92(0.22–3.86)
Retired	5	6	1.00	1.00
**Monthly Income (USD)**				
<35	78	66	1.00	1.00
35–82	47	90	2.26(1.40–3.66)	2.21(1.33–3.67)**
82–177	59	115	2.30(1.46–3.63)	1.88(1.14–3.10) *
>177	31	76	2.89(1.70–4.93)	1.92(1.03–3.57)*
**Previous eye examination**				
Yes	64	137	1.54(1.07–2.21)	1.53(1.02–2.31)*
No	151	210	1.00	1.00
**Previous cataract diagnosis**				
Yes	10	22	1.39(0.64–2.99)	0.63(0.26–1.51)
No	205	325	1.00	1.00
**Family history of cataract**				
Yes	25	62	1.65(1.00–2.72)	1.76(1.03–3.01) *
No	190	285	1.00	1.00

* p- value<0.05

** p-value <0.01

## Discussions

Favorable knowledge about cataract reduces the burden of the blindness due to cataract since it helps them to know how to delay the occurrence of the disease and initiates to take timely interventions [12, 14, 15]. In this study, 61.7% of the participants had good knowledge about cataract. Even if majority participants (more than 60%) had encouraging knowledge about symptoms, complications, curability and treatment modalities, most of them (>60%) had knowledge gap concerning to the risk factors and the prevention strategies. About 36.8% of the participants reported that their main source of information was their family/friends followed by medical personnel 32%. It was also found that good knowledge was noted in participants having higher educational level, higher income, previous eye examination and family history of cataract.

The overall knowledge found in the current study was higher than a study done in Nigeria 18.5% [22], Southern India 15% [17] and Australia (15%) [18]. The first study was conducted in rural area of South Eastern Nigeria. The second study was conducted on participants aged >15 years. The target population for the last study was age ≥49 years old. Hence, there was discrepancy with respect to target population and setting between the previous studies and the current one. This might be the reason for existed difference.

However, this result is lower than the studies done at Australia (74%)[16], Iran[26], China (70.9%)[27] and Nepal (70.4%)[14]. These studies were community-based and their target population was the age of 40 years and above except the study done in China, which was an institution based and targeted on adult aged 30 years and above. The difference might be due to the socio-demographic variance between countries as knowledge was influenced by the educational level and economic status of participants[28]. Another reason for discrepancy might be the difference in target population and study setting.

Participants who had educational level of college/university were 2.9 times more likely to have good knowledge than those who can’t read and write. This finding is supported by studies done in Canada [7], India[17] and China [29]. This might be due to the reason that when peoples educational level increase they will have great concern about their health[30]. They will also have a good attitude towards the healthcare service. Furthermore, individuals with higher educational level would read more and use social media so they would become more knowledgeable [17].

Participants with family monthly income of greater than 35USD showed a positive relationship to have good knowledge than those having <35USD monthly family income. This result was in agreement with the studies done in Iran[26], China[19] and Bangladesh[13]. This may be due to that people with better economic level would have an opportunity to access eye care service[28]. In addition, those with lower income level may fear to go to eye care center as they assume it costs a lot. However, it was not significant in studies at southern India[17].

Participants who had previous history of eye examination were 1.5 times more likely to have good knowledge about cataract than those who didn’t have eye cheek up. This might be due to the reasons that as individuals had eye examination, they may get more information from the eye care practitioners or from other patients or attendants.

Participants with a family history of cataract had 1.8 times more likely to have good knowledge than those who didn’t have. This result was in line with a study conducted in Malaya[31]. The reason for this might be that people may get more information from their parents.

As a matter of fact, the study had some limitation such as poor accessibility of street adults and adults in firms. In addition, since the study design is cross sectional it is exposed to recall bias.

## Conclusion

Significant portion of the participants had good knowledge about cataract, which was positively associated with higher level of education, higher family monthly income, presence of previous eye examination and positive family history of cataract. However, significant knowledge gap regarding the risk factors and prevention strategies was recognized. Hence, it might be logical to pay special attention how to prevent the occurrence of the disease. So, it is recommended for national and regional ministry of health offices to organize different health education programs focusing on risk factors and different prevention methods to delay occurrence of the disease. It is also recommended for researchers to conduct further similar studies in rural districts and consider different methods to include street adults and adults in firms to get more generalizable result.

## Supporting information

S1 File Pre-tested and structured questioners for the knowledge about cataract and associated factors in Gondar town, Northwest Ethiopia. (DOCX)
