# Supplementary material for: Knowledge about cataract and associated factors among adults in Gondar town, northwest Ethiopia
Source: PLoS One. 2019 Apr 23;14(4):e0215809. doi: 10.1371/journal.pone.0215809 (PMC6478322; doi:10.1371/journal.pone.0215809)
Supplement: S1 File — (DOCX) [file pone.0215809.s001.docx]

**English version of questionnaire**

Pre-tested and structured questioners for the knowledge about cataract and associated factors in Gondar town, North West Ethiopia.

Code_________ Kebele _____________

**Part 1: socio-demographic characteristics**

| Sno. | Question | Answer |
| --- | --- | --- |
| 1 | Age in years | ---------------- |
| 2 | Sex | 1. Male 2. Female |
| 3 | Religion | 1. Orthodox 2. Muslim 3. Protestant 4. Catholic 5. Other(specify)------------ |
| 4 | Marital status | 1. Married 2. Single 3. Divorced 4. Widowed |
| 5 | Educational level | 1. Cannot read and write 2. Can read and write 3. 1-8 grad 4. 9-12 grad 5. College or university |
| 6 | Occupation | 1. Civil servant 2. Farmer 3. Merchant 4. Student 5. House wife 6. Retired 7. Other Specify------------ |
| 7 | Family monthly Income | -------------------ETB |

**Part 2: previous ocular history and awareness questions**

| 1 | Did you have previous history of eye examination? | 1. Yes 2. No   If yes, continue to the next question if no go to Q no 4 |
| --- | --- | --- |
| 2 | Do you have regular eye checkup? | 1. Every 2 years or less 2. Greater than 2 years 3. When feeling pain |
| 3 | When was your last eye examination? | 1. Before 2 years 2. More than 2 years |
| 4 | Have you ever heard about cataract? | 1. Yes, If yes go to the next question no 5 2. No, **If no stop the interview hear** |
| 5 | From the following items which one is the main Source of your information? | 1. Medical personnel 2. Radio 3. Television 4. Magazines/books 5. Family/friends 6. Other specify …….. |
| 6 | Did you have previous history of cataract? | 1. Yes 2. No |
| 7 | Do you have any family who have cataract? | 1. Yes 2. No 3. I don’t know |

**Part 3: knowledge questions about cataract**

| 1 | Give simple definition of cataract | 1. Opacity on the cornea 2. A lens change where lens becomes opaque/can’t transmit light 3. A white membrane growing over the eye 4. Other specify------- 5. I don’t know |
| --- | --- | --- |
| 2 | What is/are the symptom of cataract? | 1. Pain on the eye 2. Reduction/blurring of vision 3. Redness of the eye 4. Other specify------------ 5. I don’t know |
| 3 | Can increasing age be the risk factor for cataract? | 1. Yes 2. No 3. I don’t know |
| 4 | What is the commonest age of cataract presentation? | 1. Before 40 years 2. After 40 years 3. At any age 4. I don’t know 5. Other specify---------- |
| 5 | Can trauma to the eye be the risk factor for cataract? | 1. Yes 2. No 3. I don’t know |
| 6 | Can UV light be the risk factor for cataract? | 1. Yes 2. No 3. I don’t know |
| 7 | What is the worst effect of cataract? | 1. Blindness 2. Reduction of Vision 3. Cosmetic Problems 4. No worst effect 5. Other specify---------------- 6. I don’t know |
| 8 | Is cataract treatable? | 1. Yes 2. No 3. I don’t know |
| 9 | Which one is the best treatment option for cataract? | 1. Holy water 2. Use of traditional medicine 3. Ocular medications/drops 4. Surgery 5. Other specify-------------- 6. I don’t know |
| 10 | Is it possible to get back vision from cataract blindness? | 1. Yes 2. No 3. I don’t know |
| 11 | Can we delay the onset of cataract by preventing risk factors? | 1. Yes 2. No 3. I don’t know , if yes go to the next question |
| 12 | If the answer is yes for question no 11, How? | 1. protecting the eye from injury like using eye glass 2. properly washing the eye 3. cleaning the environment 4. other specify------- 5. I don’t know |

**Amharic version of questionnaire**

ኮድ-------- ቀበሌ------------

**1. የማህበራዊ ጥያቄዎች**

| ተ.ቁ. | ጥያቄ | መልስ |
| --- | --- | --- |
| 1 | እድሜ በአመት | ---------------- |
| 2 | ፆታ | 1. ወንድ 2. ሴት |
| 3 | ሃይማኖት | 1. ኦርቶዶክስ 2. ሙስሊም 3. ፕሮቴስታንት 4. ካቶሊክ 5. ሌላ (ይጠቀስ)------------ |
| 4 | የጋብቻ ሁኔታ | 1. ያገባ/ች 2. ያላገባ/ች 3. የፈታ/ች 4. የሞተባት/የሞተችበት |
| 5 | የትምህርት ደረጃ | 1. ማንበብ እና መጻፍ የማይችል 2. ማንበብና መጻፍየ ሚችል 3. እስከ 8ተኛክፍል 4. 9-12 ክፍል 5. ኮሌጅ/ዩንቨርሲቲ |
| 6 | የስራ ሁኔታ | - - - 1. የመንግስት ሰራተኛ       2. ግብርና       3. ነጋዴ       4. ተማሪ       5. የቤትእመቤት       6. ጡሮታ       7. ሌላ ካለ ይጠቀስ------------------------------------ |
| 7 | የቤተሰብ ወርሀዊ የገቢ መጠን | --------------ብር |

1. **ከዚህ በፊት ስለተደረጉ የአይን ምርመራዎችና ስለአይንሞራ ግርዶሽ የግንዛቤ ጥያቄዎች**

| 1 | ከዚህ በፊት አየንዎትን ተመርምርው ያውቃሉ? | 1. 1. አዎ 2. የለም   መልሱ አዎ ከሆነ ወደ ቀጣዩ ጥያቄ ይለፉ፤ የለም ከሆነ ወደቁ **4** |
| --- | --- | --- |
| 2 | በየስንት ጊዜው ነው የሚመረመሩት? | 1. በሁለትአመትውስጥ 2. ከሁለት አመት ይበልጣል 3. ህመም ሲሰማኝ |
| 3 | በቅርቡ ምርመራ ያደረጉት መቸ ነበር? | 1. ከሁለት አመት በፊት 2. ከሁለት አመት በላይ ይሆናል |
| 4 | ስለ አይን ሞራ ግርዶሽ ሰምተው ያውቃሉ? | 1. አዎ አዎካሉ ወደ ሌሎች ጥያቄዎች ይልፉ 2. አላውቅም **አላውቅም ካሉ መጠይቁ ከዚህ ላይ ይቁም** |
| 5 | መረጃውን በዋናነት ከየት አገኙት ? | - - - 1. ከጤናባለሙያ       2. ከሬድዮ       3. ከቴልቭዥን       4. ከመጽሀፍ/ጋዜጣ       5. ከቤተሰብ/ጎደኛ       6. .ሌላ ካለ ይጥቀሱ------- |
| 6 | የአይን ሞራ ግርዶሽ እንዳለብዎ ተነግረው ያውቃሉ? | 1. አዎ 2. የለም |
| 7 | ከቤተሰብዎ ውስጥ የአይን ሞራ ግርዶሽ ያለበት አለ? | 1. አለ 2. የለም 3. አላውቅም |

1. **ስለአይንሞራየእውቀትጥያቄዎች**

| 1 | የአይን ሞራ ግርዶሽ ምንድን ነው? | - - - 1. የአይን ብሌን ነጭ ጠባሳ ሲፈጥር ነው       2. የአይን ሌንስ ብርሀን አለማስተላለፍ ነው       3. ነጭ መሳይ ስጋ አይን ላይ ሲያድግ ነው       4. ሌላ ካለ ይጠቀስ--------------       5. አላውቅም |
| --- | --- | --- |
| 2 | የአይን ሞራ ግርዶሽ ስሜቱ ምንድን ነው? | 1. የአይን ህመም /ውዝዋዜ 2. የእይታመቀነስ /ጉምመምሰል 3. የአይን መቅላት 4. ሌላ ካለ ይጥቀሱ 5. አላውቅም |
| 3 | የእድሜ መጨመር ለአይን ሞራ ግርዶሽ መንስኤ ሊሆን ይችላልን? | 1. ይችላል 2. አይችልም 3. አላውቅም |
| 4 | የአይን ሞራ ግርዶሽ በአብዛኛው የሚከሰተው በየትኛው የእድሜ ክልል ነው? | 1. ከ40 አመት በፊት 2. ከ40 አመት በኃላ 3. በማንኛውም ጊዜ 4. አላውቅም 5. .ሌላ (ይጠቀስ)-------------- |
| 5 | የአይን ምት ወይም ዱላ ለአይን ሞራ ግርዶሽ መንስኤ ሊሆን ይችላልን? | 1. ይችላል 2. አይችልም 3. አላውቅም |
| 6 | የፀሀይ ጨረር ለአይን ሞራግርዶሽ መንስኤ ሊሆን ይችላልን? | 1. ይችላል 2. አይችልም 3. አላውቅም |
| 7 | የአይን ሞራ ግርዶሽ የከፋ ጉዳቱ ምንድን ነው? | 1. አይነ ስውርነት 2. እይታ መቀነስ 3. ውበት መቀነስ 4. ምንም ጉዳት የለውም 5. ሌላ (ይጠቀስ)-------------- 6. አላውቅም |
| 8 | የአይን ሞራ ግርዶሽ ሊድን ይላልን? | 1. አዎ 2. ሊድን አይችልም 3. አላውቅም |
| 9 | ለአይን ሞራ ግርዶሽ የተሻለው መፍትሄ ምንድን ነው? | 1. ጠበል 2. የባህልህክምና 3. የአይን ጠብታ/መድሃኒት 4. ቀዶጥገና 5. ሌላ ካለ ይጠቀስ------ 6. አላውቅም |
| 10 | በአይን ሞራ ግርዶሽ ምክንያት ሊጠፋያ ያለን እይታ መመለስ ይቻላልን? | 1. ይቻላል 2. አይቻልም 3. አላውቅም |
| 11 | የአይን ሞራ ግርዶሽ የሚከሰትበትን ጊዜ ማዘግየት/መከላከል ይቻላልን? | 1. ይቻላል ወደ ቀጣይ ጥያቄ ሂድ 2. አይቻልም 3. አላውቅም |
| 12 | የአይን ሞራ ግርዶሽን እንዴት መከላከል ይቻላል? /የሚከሰትበትን ጊዜ እንዴት ማዘግየት ይቻላል? | - - - 1. አይንን ከአደጋ መከላከያ ዘዴዎችን እንደ መነጸር የመሰሉትን መጠቀም       2. አይንን በሚገባ መታጠብ       3. የአካባቢን ንጽህና መጠበቅ       4. ልላ ካለ ይጥቀሱ------       5. አላውቅም |
